# *In Situ* Measurement of Some Soil Properties in Paddy Soil Using Visible and Near-Infrared Spectroscopy

**DOI:** 10.1371/journal.pone.0105708

**Published:** 2014-08-25

**Authors:** Ji Wenjun, Shi Zhou, Huang Jingyi, Li Shuo

**Affiliations:** 1 Institute of Agricultural Remote Sensing and Information Technology, College of Environmental and Resource Sciences, Zhejiang University, Hangzhou, China; 2 School of Biological, Earth and Environmental Science, The University of New South Wales, Kensington, Australia; 3 Zhejiang Provincial Key Laboratory of Subtropical Soil and Plant Nutrition, Zhejiang University, Hangzhou, China; National Research Council of Italy, Italy

## Abstract

In situ measurements with visible and near-infrared spectroscopy (vis-NIR) provide an efficient way for acquiring soil information of paddy soils in the short time gap between the harvest and following rotation. The aim of this study was to evaluate its feasibility to predict a series of soil properties including organic matter (OM), organic carbon (OC), total nitrogen (TN), available nitrogen (AN), available phosphorus (AP), available potassium (AK) and pH of paddy soils in Zhejiang province, China. Firstly, the linear partial least squares regression (PLSR) was performed on the in situ spectra and the predictions were compared to those with laboratory-based recorded spectra. Then, the non-linear least-square support vector machine (LS-SVM) algorithm was carried out aiming to extract more useful information from the in situ spectra and improve predictions. Results show that in terms of OC, OM, TN, AN and pH, (i) the predictions were worse using in situ spectra compared to laboratory-based spectra with PLSR algorithm (ii) the prediction accuracy using LS-SVM (R^2^>0.75, RPD>1.90) was obviously improved with in situ vis-NIR spectra compared to PLSR algorithm, and comparable or even better than results generated using laboratory-based spectra with PLSR; (iii) in terms of AP and AK, poor predictions were obtained with in situ spectra (R^2^<0.5, RPD<1.50) either using PLSR or LS-SVM. The results highlight the use of LS-SVM for in situ vis-NIR spectroscopic estimation of soil properties of paddy soils.

## Introduction

Paddy soil is one of the most important soil resources for humans because more than half of the world's population takes rice, the typical farming product of paddy soils, as staple food. As one of the major rice producers, China has a large area of paddy fields of more than 25 million hectares, accounting for 29% of the cultivated lands of China and 23% of the world [1]. In the past 30 years, due to over-fertilization, significantly declined soil pH has been found in major crop production areas and enhanced nitrogen deposition has been identified in terrestrial and aquatic ecosystems as well as in rice [2], [3]. As a result, characterizing the properties of paddy soils in an efficient way is of great importance for management of crop growth and yield.

Over the past decades, various agricultural sensors have been used to determine the soil properties as well as their spatial variabilities [4]. Among the agricultural sensors, visible and near-infrared (vis-NIR) spectroscopy has received popularity because it is fast, less labor-intensive and cost-effective compared to conventional chemistry experiments and enables rapid measurements of various soil physical and chemical properties. However, the flooded soil condition in paddy fields makes it difficult to perform soil sampling and analysis. The best time for soil measurement is the short time gap between the harvest and following rotation, when irrigation water has been drained away. Despite the success of predicting various soil properties using laboratory-based measurement with vis-NIR spectra, the pre-treatment of samples (e.g. air-drying, grinding and sieving) is still tedious and time-consuming. With its faster and more effective characteristics compared to the laboratory-based spectroscopic measurement, in situ vis-NIR is a promising method in measuring and mapping soil properties of paddy fields [5].

Researchers have reported successful application of in situ vis-NIR spectroscopy to prediction of several soil properties. In terms of predicting clay content, Waiser *et al.* (2007) [6] found that in situ vis-NIR sensing can obtain similar results compared with laboratory-based sensing. With regard to soil organic and inorganic carbon, Morgan *et al.* (2009) [7] got slightly larger prediction errors using field-based vis-NIR measurements than using laboratory-based sensing method. When predicting soil color and mineral composition, Viscarra Rossel (2009) [8] concluded that results from in situ vis-NIR measurements were in good agreement with Munsell Book and X-ray diffraction methods. Furthermore, Mouazen *et al.* (2009) [9] improved prediction accuracy of available P by optimizing the field-based vis-NIR sensing system. In addition, a few other soil properties have been predicted with acceptable accuracy, including soil organic matter [10], nitrogen [11], [12], pH [13] and water content [12], [14]. However, most of the studies on predicting soil properties using in situ vis-NIR spectroscopy were conducted on dry farming land.

Although a couple of studies conducted on determining properties of paddy soils based on laboratory-based vis-NIR spectroscopy [15], [16], to the best of our knowledge, there are few papers published describing the systematic use of in situ vis-NIR measurements to predict soil properties in paddy fields.

The aims of this study were to evaluate the feasibility of in situ vis-NIR sensing for prediction of soil properties in paddy soils by (i) predicting various soil properties of paddy soils (i.e. organic carbon (OC), organic matter (OM), total nitrogen (TN), available nitrogen (AN), available phosphorus (AP), available potassium (AK) and pH using in situ vis-NIR spectroscopy; (ii) comparing the prediction accuracy between in situ vis-NIR spectra and laboratory-based spectra for paddy soils; (iii) evaluating the prediction accuracy of in situ vis-NIR measurements of soil properties by implementing a multivariate calibration algorithm, i.e., linear partial least square regression (PLSR), and a data-mining algorithm, i.e., least-square support vector machine (LS-SVM).

## Materials and Methods

### Ethics Statement

We randomly chose 11 paddy fields from close vicinity to 6 cities in Zhejiang province and got permission from Agricultural Bureaus from these six cities, i.e. Tonglu (2 fields, 16 samples), Jiande (2 fields, 11 samples), Pujiang (1 field, 8 samples), Zhuji (1 field, 8 samples), Yiwu (1 field, 24 samples) and Fuyang(4 fields, 117 samples). Three of the four fields we chose in Fuyang were the experimental fields in the China National Rice Research Institute. There is no endangered or protected species involved.

### Soil sampling and spectroscopic measurements

In this study, the spectra of the soil samples were recorded by proximal in situ stationary vis-NIR sensing and by laboratory-based vis-NIR measurements. A total of 184 sampling sites were randomly selected in eleven paddy fields in Zhejiang Province, China, with latitudes ranging from 29°03′N to 30°10′N, and longitudes from 119°10′E to 122°48′E. The water in the paddy fields was drained and left to dry for 10 days prior to sampling and vis-NIR measurement.

vis-NIR measurements at 104 sampling sites were taken in November 2011, while the remaining 80 sites were surveyed in August 2013. At each site, the water content of the surface soil (i.e. 0–20 cm) was firstly measured using a TDR-300 (Spectrum Technologies Inc., USA) with a 20-cm guide. Then, a soil sample was collected using a cube soil sampler to a depth of 20 cm. The surface of the sample profile was flattened and evened, without smearing the soil. Spectra were recorded at three randomly selected locations at different depths within A horizon. If there were stones, roots or voids within the soil sample, spectroscopic measurements were made on the adjacent area. For each of the three sensing locations, 10 spectra were recorded, and the mean value of the whole 30 spectra was used to represent the spectra of the soil at that site. In total, 184 spectra were recorded under the field condition with one spectrum per site.

After in situ vis-NIR measurements, the samples were packed into plastic bags, labeled and transported to laboratory. The soil samples were air-dried, ground and sieved to less than 2 mm. The vis-NIR spectra of these 184 samples were then measured again under laboratory condition. The chemical analyses of soil properties were also conducted using these samples, which would be described later.

A Fieldspec ProFR vis–NIR spectrometer (Analytical Spectral Devices, Boulder, CO, USA) was used for in situ and laboratory-based measurements. The instrument measures the spectra between 350 and 2500 nm, with a resolution of 3 nm at 700 nm and 10 nm at 1400 nm and 2100 nm. The sampling resolution of the spectra is 1 nm. To implement in situ sensing, a high intensity contact probe (Analytical Spectral Devices) was used to prevent the interference from stray light during measurement. The probe has its own light source and a viewing window of 2 cm in diameter through which the measurements are made. To keep the measurement consistent, the contact probe was also used in the laboratory-based measurement. A Spectralon panel with 99% reflectance was used to calibrate the spectrometer before each measurement.

### Chemical analysis

Soil OM was measured using the H_2_SO_4_-K_2_Cr_2_O_7_ oxidation method at 180°C for 5 minutes [17]. Soil TC and OC content were determined by dry combustion at 1100°C with a multi N/C 3100 (Analytik Jena AG, Germany). Before the determination of soil OC, soil samples were acidized by hydrochloric acid to remove the inorganic carbon in the soil. Soil TN was measured using Semi-micro Kjeldahl Method and soil AN was measured by the alkaline hydrolysis diffusion method [18]. Soil AP was measured by the NH_4_F-HCl method [18]. Soil AK was measured using the NH_4_OAC extraction method and analyzed using a flame photometer [18]. Soil pH was measured in a 1∶1 soil: water suspension [18]. The statistics of measured soil properties are listed in Table 1.

**Table 1:** Statistics of paddy soil samples in this study.

Soil property	Unit	Dataset	NO. samples	Mean	St.Dev	Medium	Max	Min
OC	g/kg	All	183	15.87	6.65	14.53	36.29	4.12
		Training	138	15.89	6.73	14.51	36.29	4.12
		test	45	15.81	6.48	14.53	35.26	4.78
OM	g/kg	All	104	29.1	13.7	25.9	60.5	6.9
		Training	78	28.9	13.6	25.8	60.5	6.9
		test	26	29.8	14.0	26.6	60.5	7.4
TN	%	All	104	0.17	0.08	0.15	0.37	0.03
		Training	78	0.16	0.08	0.15	0.37	0.03
		test	26	0.17	0.09	0.15	0.36	0.04
AN	mg/kg	All	104	128.43	60.44	132.00	295.00	15.80
		Training	78	127.59	60.37	132.00	295.00	15.80
		test	26	130.96	61.77	132.50	264.00	18.00
AP	mg/kg	All	104	22.86	18.37	18.90	108.00	0.70
		Training	78	22.65	18.52	18.55	108.00	0.70
		test	26	23.48	18.26	19.65	75.60	2.00
AK	mg/kg	All	104	56.75	15.56	55.10	105.00	32.50
		Training	78	55.48	15.51	55.05	105.00	32.50
		test	26	57.55	15.99	55.35	97.30	34.60
pH	-	All	104	5.74	1.17	5.20	8.43	4.60
		Training	78	5.73	1.17	5.19	8.43	4.60
		test	26	5.79	1.21	5.22	8.32	4.62

### Data pre-processing

The spectral regions for 350–399 nm and 2451–2500 nm were deleted because of noise. The reflectance spectra were transformed to apparent absorbance (log1/R) and then mean centered. The smoothing process of the spectra was made using the Savitzky-Golay algorithm with a window size of 11 and polynomial of order 2 [19]. One sample was regarded as outlier and removed from the dataset because its spectra were strange. For each soil property, corresponding values were sorted from small to large, and then every forth one was selected into test dataset, leaving the rest in training dataset.

### Partial least square regression (PLSR)

Among the multiple linear calibration algorithms, partial least square regression (PLSR) [20] is one of the most popular algorithms used for spectral calibration and prediction. It is closely related to principal component regression (PCR) yet with a slight difference. Both of them compress the data before prediction while PLSR avoids the dilemma encountered by PCR of choosing components for the regression [21].

We assume the spectral data matrix used as independent variable into PLSR is ***X***, where ***X*** = [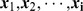], and soil properties as dependent variable is *y*, with both mean-centered. The first step to perform PLSR is to extract a few linear combinations (called components or factors), ***T***, of the original spectral matrix ***X***:
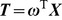
(1)where 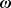 are the scaled weights and can be calculated as the eigenvectors of the matrix 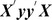. Then both ***X*** and *y* can be regressed onto ***T*** as follows:
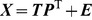
(2)

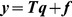
(3)where ***P*** are spectral loadings and *q* are chemical loadings, describing how the variables in ***T*** relate to ***X*** and ***y***. ***E*** and ***f*** are residuals and represent noise or irrelevant variability in ***X*** and ***y***. After the model parameters are estimated, they can be combined into the final prediction model as
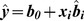
(4)where ***b_0_*** is the intercept and 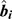 are regression vectors. The detailed description of 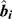 can be found in the book of [21].

To avoid over-fitting or under-fitting, leave-one-out cross validation was used to determine the number of factors to retain in the calibration models [22]. Root mean square error of cross validation (RMSE) and Akaike information criterion (AIC) [23] were used to decide the number of factors.
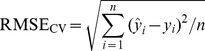
(5)


Where 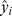 is the predicted value and 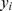 is the observed value, n is the number of calibration samples.
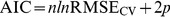
(6)


Where n is the number of samples and *p* is the number of features used in the prediction. The best model has the smallest
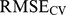
and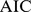.

### Least square support vector machine (LS-SVM)

Support vector machine (SVM) is a kernel-based learning algorithm [24] and has been widely used in the pattern classification and regression. The kernel-based learning methods use an implicit mapping of the input data in a high dimensional feature space, a special type of hyperplane defined by a kernel function, in which a regression model is built. As an optimized algorithm based on standard SVM, the least-squares support vector machine (LS-SVM) [25] uses a squared loss function instead of the e-insensitive loss function, from which equality constraints rather than inequality constraints follow. Compared to SVM, complex calculations are avoided in LS-SVM and the multivariate calibration problem can be solved in a relatively fast way. The theory of LS-SVM has been introduced by Suykens *et al.* (2002) [25].

Similarly, the spectral data matrix used as independent variable is ***X***, where ***X*** = [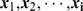], and soil properties as dependent variable is *y*. The LS-SVM uses nonlinear regression function:



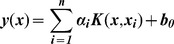
(7)where 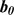 is the bias; n is the number of samples; 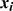 is the measured vis-NIR spectra of different samples; 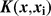 is defined by the kernel function. We used radial basis function kernel (RBF), which is the typical general-purpose kernel:
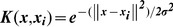
(8)where σ^2^ is the RBF kernel function parameter, determining the width of the kernel.


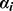
 is Lagrange multipliers (i.e. support value), which is used by solving the linear Karush-Kuhn-Tucker (KKT) system:
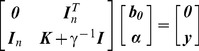
(9)where 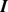 refers to an (n×n) identity matrix; γ is the regularization parameter which balances the model's complexity and the training errors; 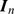 is a (n×1) vector, with all elements ones; ***y*** is an (n×1) vector of observed properties values and 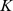 denotes elements in kernel matrix.

As we can see from these formulas, in order to make an LS-SVM model, two additional parameters (i.e. γ and σ^2^) need to be determined by users. The regularization parameter γ determines the trade-off between the fitting error minimization and smoothness of the estimated function, and is important to improve the generalization performance of the LS-SVM model. An increase in γ is analogous to an increase in the number of latent variables in a PLS model [26]. The RBF kernel function parameter σ^2^ changes the width of the kernel, and thus the degree of the non-linearity can be modeled. When σ^2^ increases, the kernel becomes confined, forcing the model towards a linear regression, and its accuracy decreases as well. By contrast, decreased σ^2^ and increased γ may lead to over-fit and thus should be treated cautiously [26].

### Assessment of statistics

Coefficients of determination (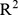), root mean square error (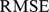) and the ratio of prediction derivation (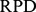) were used to compare the prediction accuracies.
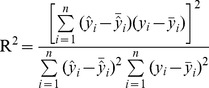
(10)

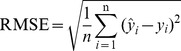
(11)

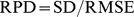
(12)


Where 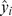 is the predicted value and 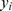 is the observed value; 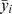 is the mean of observed value; 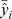
is the mean of predicted value; SD is standard deviation of observed values; *n* is the number samples.

Williams (2003) [27] and Saeys *et al.* (2005) [28] proposed a criterion for the classification of R^2^ and RPD: an R^2^ value below 0.50 or an RPD value below 1.5 indicates very poor model predictions and such a value could not be useful; an R^2^ value between 0.50 and 0.65 an RPD value between 1.5 and 2.0 indicates a possibility of distinguishing between large and small values, while an R^2^ value between 0.66 and 0.81 or an RPD value between 2.0 and 2.5 makes approximate quantitative predictions possible. For an R^2^ value between 0.82 and 0.90 or an RPD value between 2.5 and 3.0 and above 3.0, the prediction is classified as good. If R^2^ value is larger than 0.91 and RPD value is larger than 3.0, the prediction is considered excellent. Generally, a good model prediction would have large values of R^2^ and RPD, and a small value of RMSE. In order to simplify the classification, Grade A to E was assigned to the accuracy classes from excellent to not useful.

The LS-SVM toolbox (LS-SVM v.1.5, Suykens, Leuven, Belgium) was applied with Matlab R2009 (MathWorks, Inc., Natick, MA) to perform the LS-SVM models. And other data analysis was conducted in R 2.15.0 [29].

## Results and Discussions

### Comparison of in situ spectra and laboratory-based spectra

**Figure 1:**
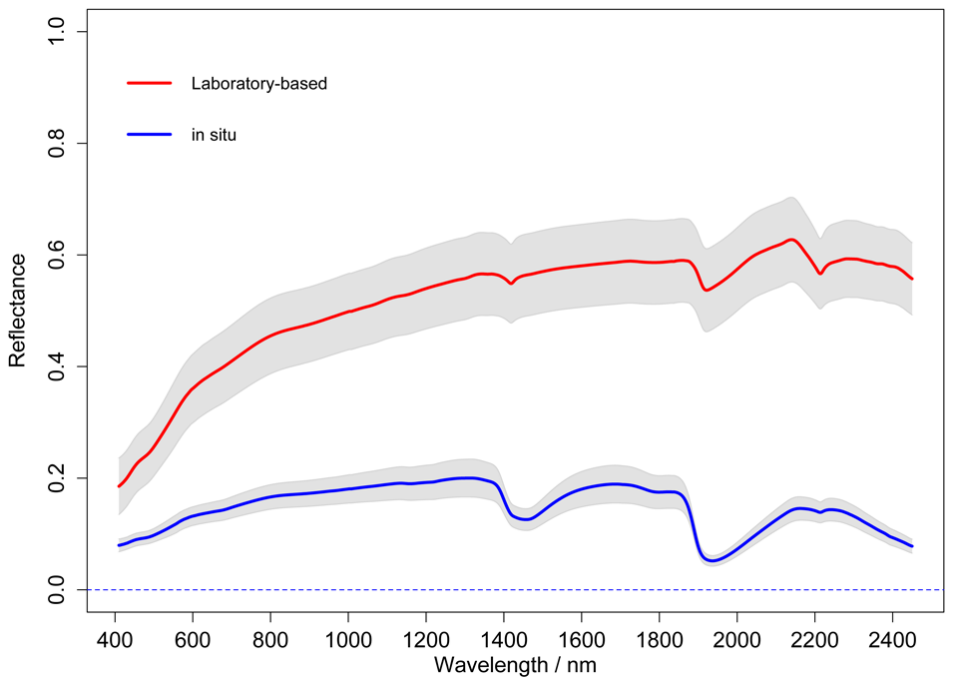
The average reflectance spectra measured in laboratory (red) and in situ (blue) and their corresponding standard deviation values (shaded regions).

The average reflectance (R) of in situ and laboratory-based measurements of 183 samples and their respective standard deviations are given in Fig. 1. In brief, in situ spectra have smaller reflectance values compared with the laboratory-based spectra. This is because the presence of soil moisture, replacing the air within the soil gaps, increases forward scattering of light and thus the whole absorption of soil moisture at each wavelength increases [30].

**Figure 2:**
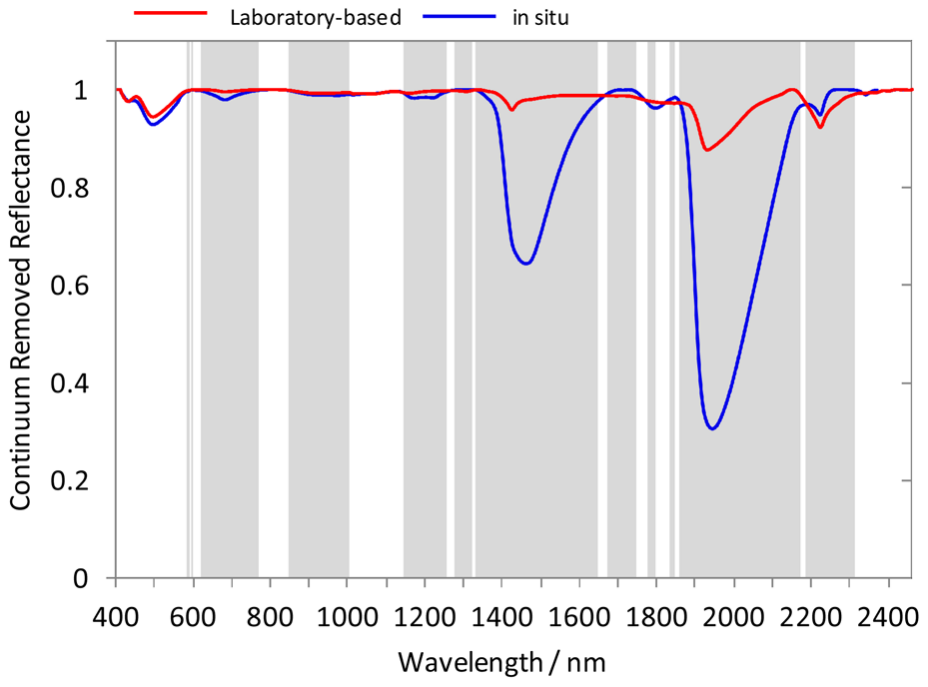
Wavelength specific t-tests between continuum removed laboratory-based and in situ spectra. Note: The shaded regions show where significant differences occur between the spectra at 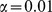
significance level.

Near Infrared (NIR) spectra are dominated by weak overtones and combinations of fundamental vibration which occurs in the MIR region, while visible spectra mainly comprise of electronic transitions [22]. The absorption features of the raw reflectance spectra are usually broad and weak and some of them are difficult to distinguish with the naked eye. As such, continuum removal was applied to all spectra to emphasize absorption features in the spectra. The averaged continuum removed reflectance (CR) is given in Fig. 2, and wavelength specific t-tests were performed between the continuum removed laboratory-based and in situ spectra. In Fig. 2, shaded regions show where there were significant differences between the spectra at 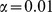 significance level. The absorption features due to soil iron oxides near 430 nm and 480 nm [31] have similar size and shape in both in situ and laboratory-based spectra. However, the absorption feature near 650 nm probably correlated with haematite (Fe_2_O_3_) [32], [33] of in situ spectra is greater than that of laboratory-based measurements. The shallow absorption near 1000 nm may be due to amidogen group present in both in situ and laboratory-based spectra, and they are significantly different. The most obvious differences between the two types of spectra are located in two primary water absorption regions within the NIR spectrum, i.e. one around 1450 nm and the other near 1950 nm. It can be explained by the permanently waterlogged conditions of the paddy soil samples. The absorptions caused by soil moisture increases when soil is wet and their features broaden and deepen compared to laboratory collected spectra. However, the strong water absorption near 1950 nm of in situ field collected spectra partly masks the absorptions of clay minerals near 2200 nm which can be identified in the dry laboratory-based spectra. It might affect the prediction accuracies of the spectroscopic models [30].

### Prediction of soil properties using PLSR

**Figure 3:**
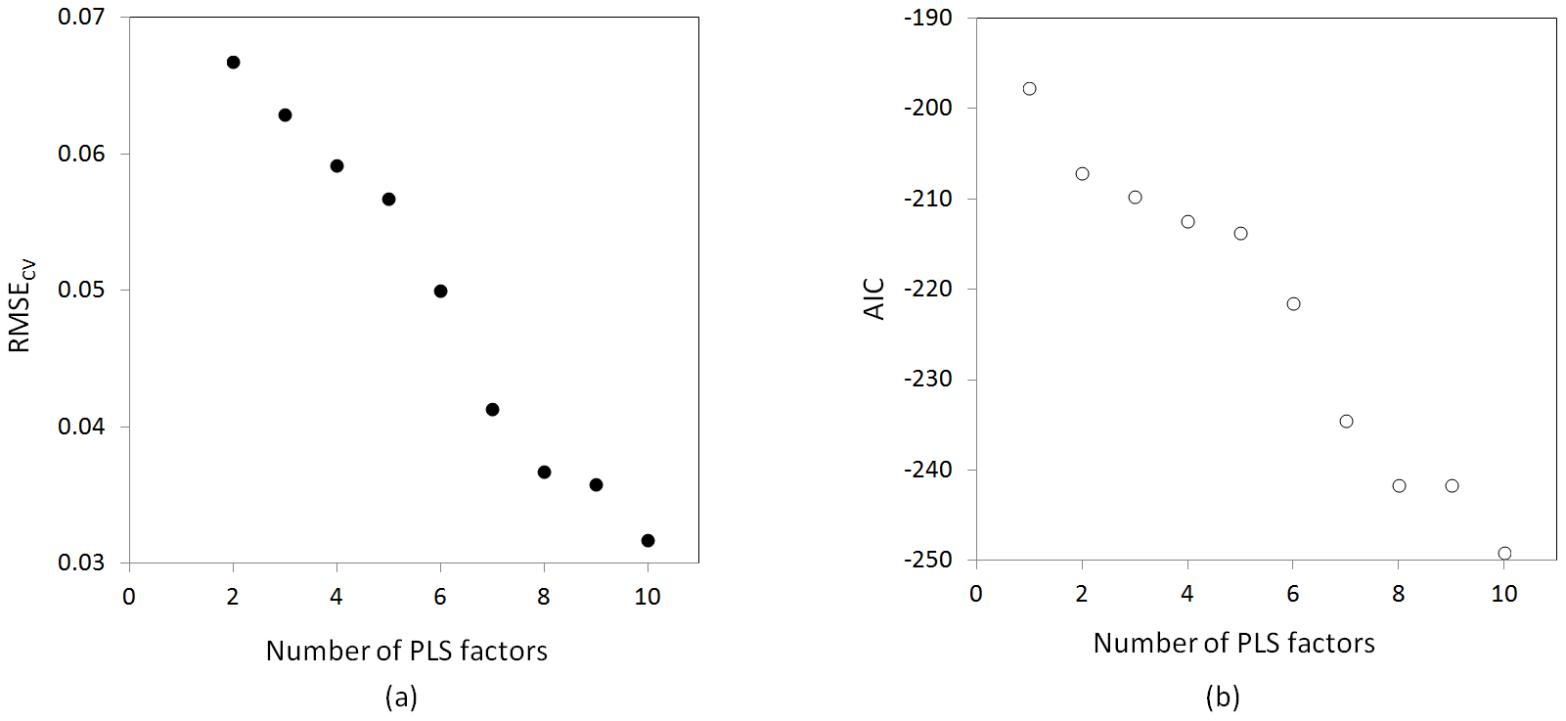
Number of factors (NF) used in partial least square regression versus (a) cross-validated root mean square error (RMSEcv) and (b) Akaike Information Criterion (AIC).

PLSR algorithms were performed on the training dataset with the optimal number of factors decided by leave-one-out cross validation, and the test dataset was used to validate the PLSR model independently. Taking TN for example, the cross-validated RMSEcv and AIC were plotted against the number of factors (Fig. 3). The optimal number of factors was selected based on the minimum RMSE_CV_ and AIC. Meanwhile, a small number of factors should be included in the model to reduce its complexity when comparable predictions can be obtained. As a result, 8 factors were selected to be used in PLSR with laboratory vis-NIR spectra.

Prediction accuracy of seven soil properties with laboratory-based soil spectra using PLSR method and their accuracy classes are presented in Table 2. Of all the measured soil properties, TN was best predicted with R^2^ of 0.87 and RPD of 2.81(Grade B). OM and OC were approximately quantitatively predicted (Grade C), with R^2^ of 0.81, RPD of 2.30 and R^2^ of 0.81, RPD of 2.20 for OM and OC, respectively. The predictions of TN, OM and OC is comparable to previous studies [34], [35] The successful predictions of these properties are mainly because carbon and nitrogen have direct spectral responses due to the overtones and combinations of N-H, C-H+C-H and C-H+C-C in the vis-NIR spectra [36], [37].

**Table 2:** Comparison of prediction accuracy for in situ PLSR, laboratory-based PLSR and in situ LS-SVM.

Soil property	Unit	in situ spectra + PLSR					Laboratory-based spectra + PLSR					in situ spectra + LS-SVM					
		No. factors	R^2^	RMSE	RPD	Grade	No. factors	R^2^	RMSE	RPD	Grade	γ	σ^2^	R^2^	RMSE	RPD	Grade
OC	g/kg	10	0.75	3.33	1.95	D	9	0.81	2.94	2.20	C	26	6346	0.79	2.95	2.20	C
OM	g/kg	10	0.75	7.66	1.83	D	8	0.81	6.11	2.30	C	559	19647	0.81	6.41	2.18	C
TN	%	8	0.86	0.03	2.68	B	8	0.87	0.03	2.81	B	32	1064	0.88	0.03	3.05	A
AN	mg/kg	8	0.76	32.41	1.91	D	8	0.86	24.76	2.49	B	171	1326	0.76	32.27	1.91	D
AP	mg/kg	4	0.43	13.71	1.33	E	8	0.29	19.33	1.17	E	2	714	0.36	14.33	1.27	E
AK	mg/kg	6	0.03	17.92	0.89	E	10	0.07	20.82	0.77	E	23	236	0.14	17.66	0.91	E
pH	Unit	9	0.77	0.58	2.11	C	8	0.82	0.51	2.42	B-C	2548	813	0.80	0.54	2.23	C

However, the prediction accuracy often varies with the forms of carbon and nitrogen present in the soils [36], [38]. The phenomenon also occurs in our results. For example, prediction of AN shows a lower accuracy than that of TN with R^2^ of 0.86 and RPD of 2.49 (Grade B). This is because most of AN in soil is inorganic, which have no characteristic absorption in vis-NIR region, and the amount of AN is usually small, generally less than 5% of TN, which have a slighter effect on soil spectra.

Although some researchers have reported successful prediction of AP and AK using vis-NIR [39]–[42], it is not the case in this study. AP was not well predicted in consideration of R^2^ of 0.29 and RPD of 1.17 (Grade E); prediction of AK was even worse with R^2^ of 0.07 and RPD of 0.77 (Grade E). It is because there is no direct spectral absorption features in the vis-NIR region for AP and AK. The occasionally successful prediction of these soil properties may be due to the covariation with other soil properties which have directly spectral responses in the vis-NIR range [37]. However, in the present study, poor correlations of AP or AK with carbon and nitrogen have been found (see Table 3).

**Table 3:** Upper triangular correlation matrix among six soil properties.

Correlations	OC	OM	TN	AN	AP	AK	pH
OC	1	0.96	0.96	0.93	0.00	0.42	−0.21
OM		1	0.98	0.93	0.06	0.45	−0.29
TN			1	0.95	0.09	0.471	−0.27
AN				1	0.12	0.41	−0.39
AP					1	−0.04	−0.46
AK						1	0.13
pH							1

Additionally, pH can be predicted with approximately quantitative accuracy with R^2^ of 0.82 and RPD of 2.42 (Grade B–C). Although without direct spectral responses in the vis-NIR region, measurements of pH were always reported to be more successful compared to P and K [43], [44]. It might be because pH is related to wavelengths of minerals [33]. However, further investigation was needed.

### Spectroscopic prediction using PLSR: in situ vs. laboratory-based

Prediction accuracies with in situ collected spectra using PLSR are given in Table 2. Compared to laboratory-based spectroscopic measurements, predictions of soil properties, such as OC, OM, TN, AN and pH, with in situ measured spectra were worse. For example, predictions of soil OM and OC using laboratory-based spectra were considered to be approximately quantitatively accuracy (Grade C) while those using in situ measurements were only able to be distinguished between high and low values (Grade D). Besides, the prediction accuracy of AN decreases to Grade D using in situ spectra (R^2^ = 0.76 and RPD = 1.91) from Grade B using laboratory-based spectra (R^2^ = 0.86 and RPD = 2.49). It may be caused by the environmental factors existing during the in situ measurement, such as soil moisture, ambient light, temperature and condition of the soil surface, which would partly mask the absorption features of some soil properties.

As prediction of soil properties with in situ vis-NIR spectra is less accurate than with laboratory-based measurement when linear calibration algorithm was used, a non-linear data mining (i.e. LS-SVM) algorithm was carried out aiming to extract more useful information from the in situ spectra and improve predictions.

### Spectroscopic prediction of soil properties: PLSR vs. LS-SVM

**Figure 4:**
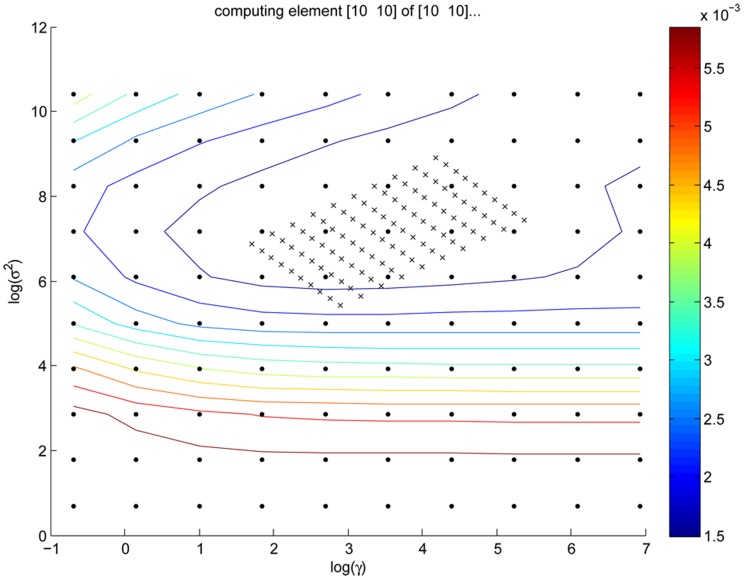
Grid search on γ and σ^2^ using least square support vector machine (LS-SVM).

In attempt to improve the prediction accuracy using in situ soil spectra, LS-SVM was used to build the models. In order to determine the parameters of γ and σ^2^ for LS-SVM models, γ ranging from 2^−1^ to 2^10^ and σ^2^ ranging from 2 to 2^15^ were tested. The ranges were based on previous studies. For each combination of γ and σ^2^, the root mean square error of cross-validation (RMSE_cv_) was calculated and the optimal parameters were determined when smaller RMSE_cv_ occurred. The optimizing process of predicting TN is shown in Fig. 4. The grid search and leave-one-out cross validation were employed to find the optimal combination of γ and σ^2^. Grid search is a two-dimensional minimization procedure based on exhaustive search in a limited range. The grids of “.” in the first step was 10×10, and the searching step at this stage was relatively large. The grids of “×” in the second step was 10×10, and the searching step in the second stage was relatively small. The optimal search area was determined using the contour lines of RMSE_cv_ plotted in Fig. 4.

**Figure 5:**
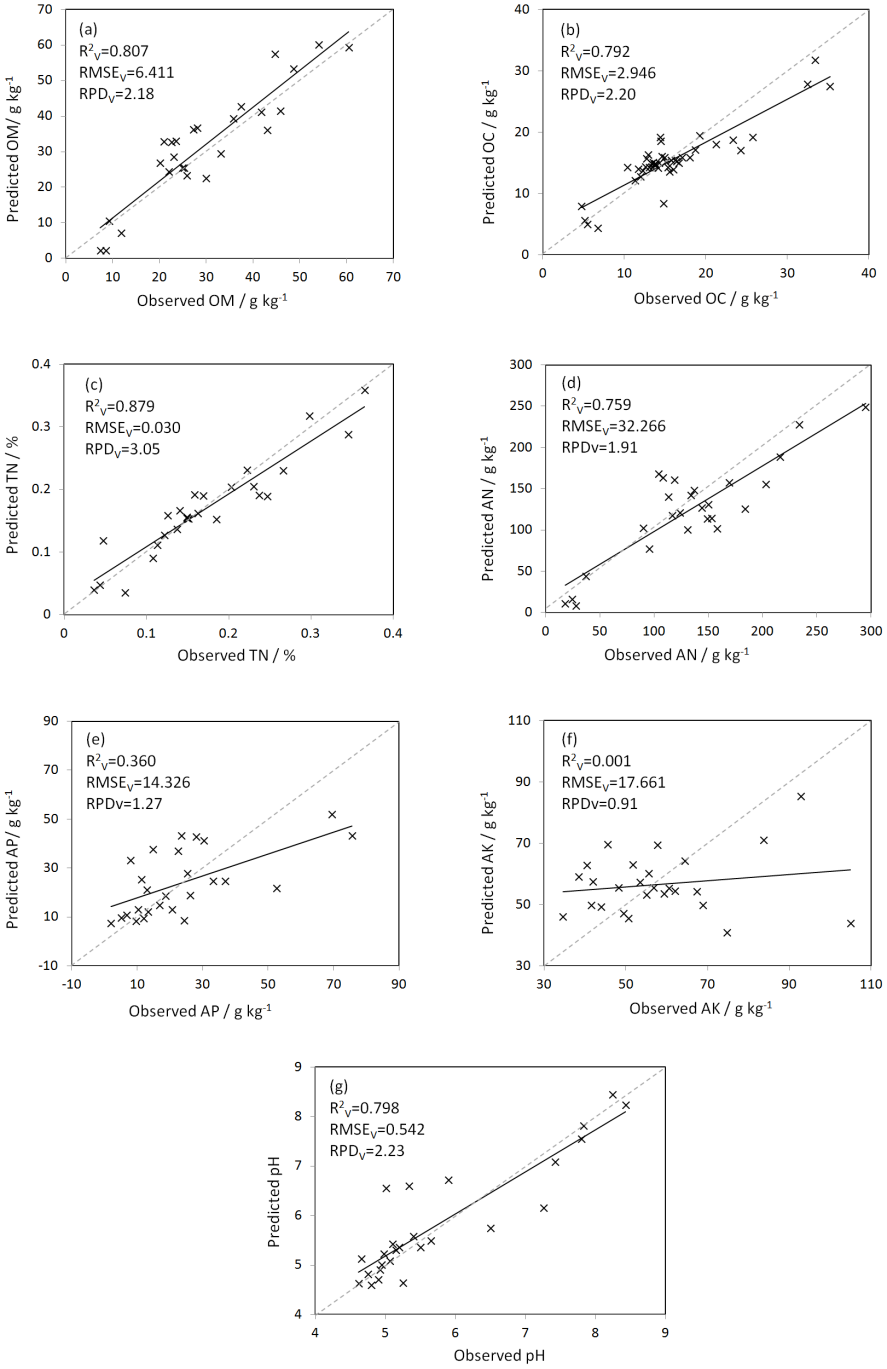
Predicted versus observed values of soil (a) OM, (b) OC, (c) TN, (d) AN, (e) AP, (f) AK, and (g) pH using least square support vector machines (LS-SVM) with in situ vis-NIR spectra.

Predictions with in situ spectra using LS-SVM can be found in Table 2. Firstly, comparison between PLSR and LS-SVM was made with in situ spectra. Soil OM and OC can only be distinguished by high and low values (i.e. Grade D) when PLSR method was performed (OM: R^2^ = 0.75 and RPD = 1.83; OC: R^2^ = 0.75 and RPD = 1.95). However, using LS-SVM method, both OM and OC can be approximately quantitatively estimated (i.e. Grade C), with the prediction accuracies of R^2^ = 0.81 and RPD = 2.18 for OM, and R^2^ = 0.79 and RPD = 2.20 for OC. Prediction of TN was even more accurate using LS-SVM with R^2^ = 0.88 and RPD = 3.05 (i.e. Grade A) compared to PLSR with R^2^ = 0.86 and RPD = 2.68 (i.e. Grade B). Besides, comparable prediction accuracies of AN were obtained between LS-SVM and PLSR, both with R^2^ = 0.76 and RPD = 1.91 (Grade D). In terms of pH, LS-SVM only slightly improved the prediction compared to PLSR. However, AK and AP remained unpredictable (Grade E) using two methods. The use of the data-mining algorithm (i.e. LS-SVM here) improved the prediction accuracy of most of soil properties compared with the linear PLSR algorithm with in situ vis-NIR spectra. Fig. 5 shows the predicted values of seven soil properties against the observed ones using LS-SVM with in situ vis-NIR spectra.

Surprisingly, the predictions of OM, OC and pH with in situ spectra using LS-SVM were comparable to those using PLSR with laboratory-based spectra; prediction of TN using in situ spectra with LS-SVM was one grade better than using laboratory-based measurement with PLSR. The prediction accuracy of TN is comparable to the result from Kleinebecker et al. (2013) [45] with air dried samples. However, in term of AN, laboratory-based model with PLSR still offers better prediction. Given the improved prediction results of OM, OC, TN and pH using LS-SVM, in situ vis-NIR spectroscopy would become an effective tool for rapid and reliable measurement of soil properties in the field.

### In *situ* prediction: paddy soils vs. irrigated soils

The prediction of paddy soil properties with in situ vis-NIR spectra were compared to a recent review of in situ vis-NIR measurements [36] of irrigated (arable) soils, i.e. dry-farming soils (Table 4). Prediction accuracy of TN and pH of paddy soils is similar to that of irrigated soils. However, due to the presence of considerable amount of soil water in paddy soils, which affects the in situ measured soil vis-NIR spectra, OC, AP and AK are better predicted in irrigated soils compared to paddy soils.

**Table 4:** Comparison of prediction accuracy of soil properties with in situ vis-NIR for paddy soils and irrigated soils (dry-farming).

Soil property	Paddy soils (PLSR)	Paddy soils (LS_SVM)	irrigated soils [36]
OC	D	C	B–C
OM	D	C	N.A.
TN	B	A	B
AN	D	D	N.A.
AP	E	E	C
AK	E	E	D
pH	C	C	C

## Conclusions

Compared with laboratory-based vis-NIR spectroscopic measurement, field-based measurement is more efficient by measuring soil spectra directly in situ. It thus offers a promising way to analysis soil properties quickly in paddy fields when water is drained away before and after harvest. In our study, systematic research on paddy soil properties using in situ vis-NIR spectra and laboratory-based vis-NIR spectroscopy were carried out, including soil organic matter (OM), total organic carbon (OC), total nitrogen (TN), available nitrogen (AN) available phosphorus (AP), available potassium (AK) and pH.

Using the PLSR algorithm with laboratory-based vis-NIR spectra, soil OM, OC, TN, AN and pH can be quantitatively estimated with various accuracies while AP and AK can be poorly predicted. However, the prediction accuracy of soil properties decreased to some extent when in situ spectra were used for modeling. It happened especially for the prediction of soil OM, OC, AN and pH, with one grade decreasing. It might be due to the existence of soil moisture and ambient light, as well as the environment temperature and soil surface condition, which might mask or partly mask the absorption information on spectra, and influence their prediction accuracies.

By performing the non-linear LS-SVM algorithm, prediction of soil OM, OC, TN and pH with in situ vis-NIR spectra was obviously improved. Their predictions were comparable or even better than laboratory-based spectroscopic measurement using PLSR algorithm. Prediction of AN was not improved and AP and AK remained unpredictable. Thus, we propose the use of LS-SVM algorithm for in situ vis-NIR spectroscopic estimation of soil properties of paddy soils.

Owing to the permanently waterlogged conditions of paddy soils, in situ prediction of several soil properties of paddy fields is less accurate compared with irrigated soils. Other data mining methods are expected to be tested on the in situ paddy soil spectra. Besides, further research on the chemometic algorithms for removing the effects of water and other environmental factors from the spectra might fundamentally improve the prediction of soil properties with in situ spectra.

## Supporting Information

File S1 In situ measured vis-NIR spectra of 184 samples. To every tenth wavelength was retained to reduce the size of the file.
(XLSX)
