# Correction: *In Situ* Measurement of Some Soil Properties in Paddy Soil Using Visible and Near-Infrared Spectroscopy

**DOI:** 10.1371/journal.pone.0159785

**Published:** 2016-07-14

**Authors:** Wenjun Ji, Zhou Shi, Jingyi Huang, Shuo Li

The authors’ names are listed incorrectly in the published article. The correct names are: Wenjun Ji, Zhou Shi, Jingyi Huang, Shuo Li. The correct citation is: Ji W, Shi Z, Huang J, Li S (2014) *In Situ* Measurement of Some Soil Properties in Paddy Soil Using Visible and Near-Infrared Spectroscopy. PLoS ONE 9(8): e105708. doi:10.1371/journal.pone.0105708

## References

[1] Wenjun J, Zhou S, Jingyi H, Shuo L (2014) *In Situ* Measurement of Some Soil Properties in Paddy Soil Using Visible and Near-Infrared Spectroscopy. PLoS ONE 9(8): e105708 doi:10.1371/journal.pone.0105708 25153132 10.1371/journal.pone.0105708 PMC4143279

